# Tarnished jewellery and skin – a subtle external marker indicating exposure to hydrogen sulfide

**DOI:** 10.1007/s12024-024-00862-z

**Published:** 2024-07-17

**Authors:** John D. Gilbert, Roger W. Byard

**Affiliations:** 1 Forensic Science SA, 21 Divett Place, Adelaide, 5000 Australia; 2 The University of Adelaide, Frome Rd, Adelaide, 5005 Australia

**Keywords:** Hydrogen sulfide, Jewellery, Skin marking, Suicide

## Abstract

A 39-year-old woman was found lying in the rear of a car at her home address. A smell of rotten eggs was noted and bottles of brick, tile and paver cleaner and lime sulfur were found in the rear of the vehicle. Suicide notes were found in the house. At autopsy there was no evidence of significant trauma with black tarnishing of a silver-coloured ring and a silver-coloured necklace noted with staining of the underlying skin, in keeping with a chemical reaction between hydrogen sulfide and silver. Internally the most significant finding was unusual greenish discolouration of the gray matter of the external and cut surfaces of the cerebral hemispheres, cerebellum and brain stem. No other organs had this discoloration. Death was attributed to hydrogen sulfide poisoning. Skin discoloration from silver jewellery may represent a subtle external marker for lethal or non-lethal hydrogen sulfide exposure.

## Case report

A 39-year-old woman with a history of anxiety and depression was found lying in the rear of a car at her home address. A sign in the window of the car warned of ‘hydrogen cyanide’ gas. A gas detector worn by emergency services responders alerted to the presence of a toxic gas. She was extricated from the vehicle and cardiopulmonary resuscitation was unsuccessfully undertaken. Death was certified at the scene. Attending police officers noted a smell of rotten eggs and bottles of brick, tile and paver cleaner and lime sulfur were found in the rear of the vehicle. Suicide notes were found in the house.

**Fig. 1:**
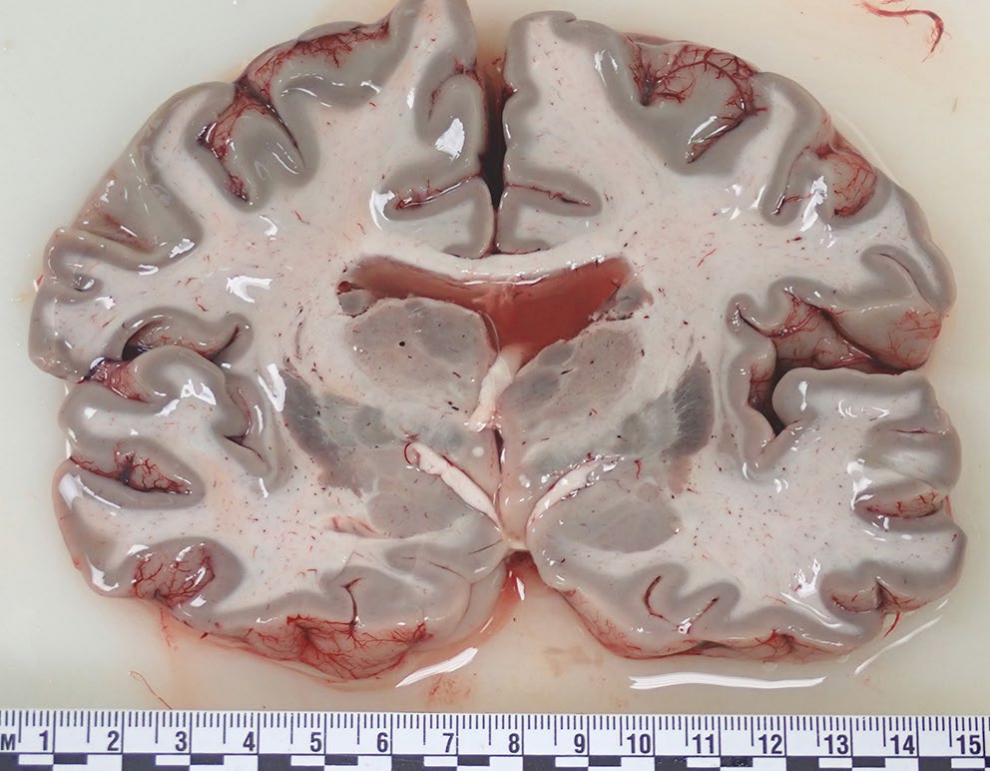
A cross section of fresh brain in a 39-year-old woman who died of self-administered hydrogen sulfide poisoning showing the characteristic greenish discoloration of the cerebral gray matter

At autopsy the internal examination showed marked pulmonary edema and congestion, soiling of the larger airways by gastric contents, and a congenitally bicuspid aortic valve with no significant stenosis. The eternal and cut surfaces of the cerebral hemispheres, cerebellum and brain stem showed a generalised unusual greenish discolouration of the gray matter (Fig. 1) with no other abnormalities. No other organs had this discoloration.

**Fig. 2:**
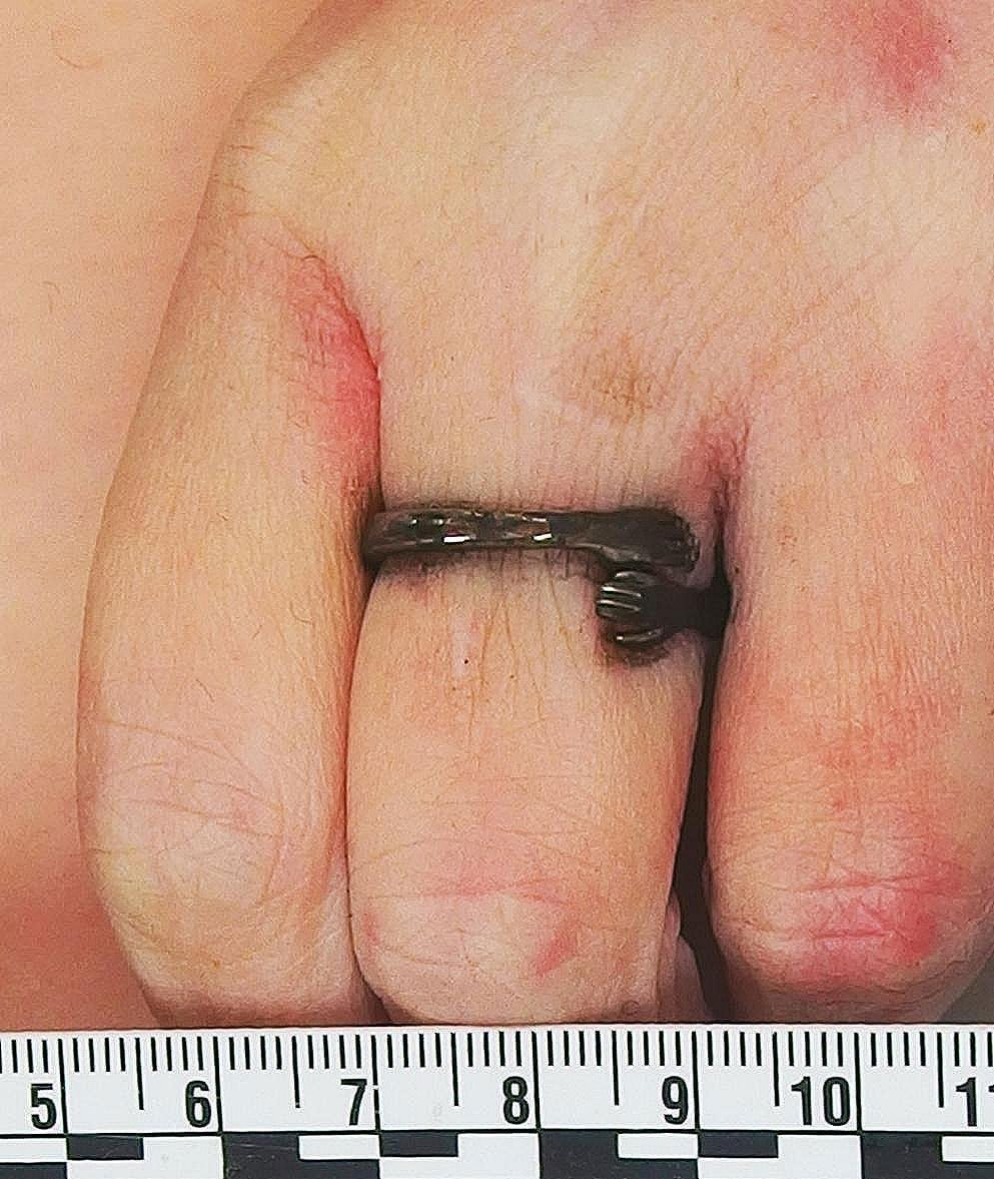
A tarnished silver ring on the left middle finger caused by a reaction with hydrogen sulfide producing silver sulfide (Ag_2_S)

**Fig. 3:**
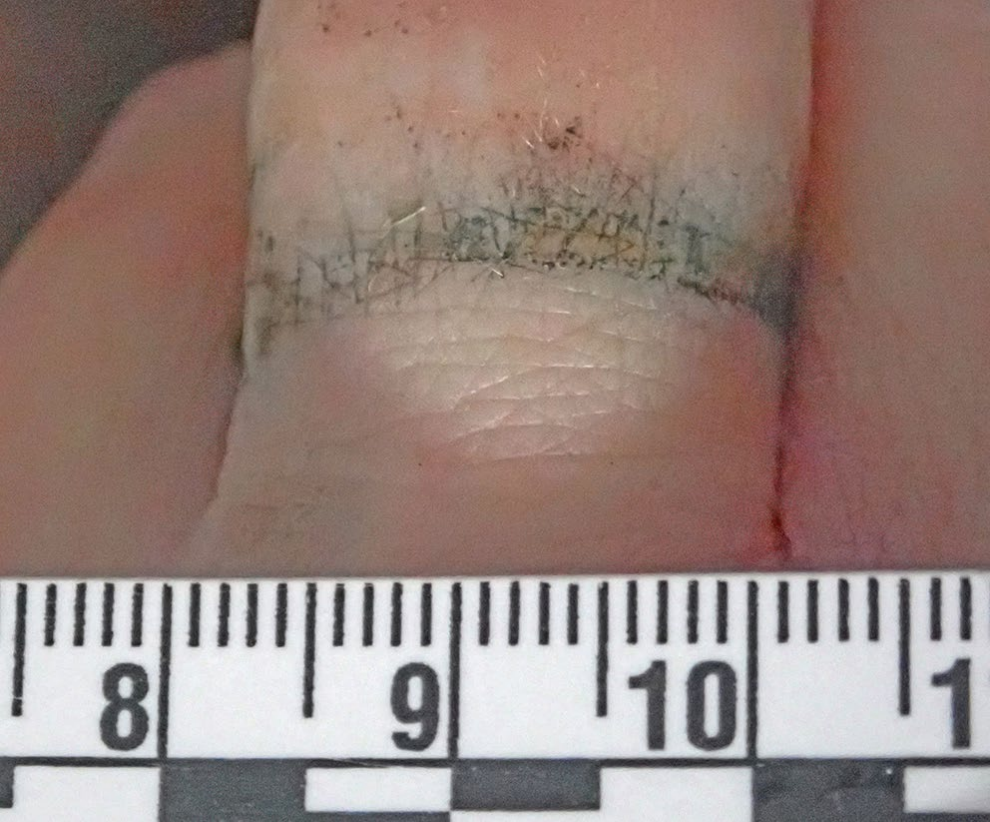
Discoloration of the underlying skin revealed after removal of the tarnished ring

External examination had shown there was no evidence of significant trauma other than multiple parallel linear scabbed superficial incised wounds over the anterior aspect of the right thigh consistent with self-infliction. An unusual finding was that of black tarnishing of a silver-coloured ring and a silver-coloured necklace with staining of the underlying skin (Figs. 2 and 3), in keeping with a chemical reaction between hydrogen sulfide (H_2_S) and silver.

Analysis of a specimen of preserved peripheral blood obtained at autopsy showed no alcohol, with a greater than therapeutic level of venlafaxine (possibly due to post mortem drug redistribution given the 7-day interval between death and autopsy), and non-toxic levels of quetiapine and diazepam. Modafanil, a drug prescribed for narcolepsy and sleep apnea, was detected but not quantitated. Although hydrogen sulfide poisoning may be supported by the detection of sulfide and thiosulfate in the blood, analytical methods for this kind of testing are unfortunately not available at our institution.

Brick, tile and paver cleaner contains sulfamic acid and hydrochloric acid and lime sulfur contains calcium polysulfides. The combination of the two will produce large volumes of hydrogen sulfide gas which has a characteristic rotten egg smell as noted by police at the scene. Hydrogen sulfide reacts with metallic silver to produce black tarnishing (silver sulfide) and causes unusual greenish discolouration of the gray matter of the brain.

Given the scene findings, the presence of relevant chemicals, the odour of rotten eggs and the autopsy findings including tarnished silver jewellery and green discolouration of the brain, the death was attributed to hydrogen sulfide poisoning. There were no injuries or other markings on the body to indicate the involvement of another person in the death, and no underlying natural diseases were present that could have caused or contributed to the death.

## Discussion

This paper is not aimed in any way at developing an understanding of hydrogen sulfide poisoning as this information is already available in the literature. Instead the purpose of the paper is to report characteristic staining of the skin from the interaction of hydrogen sulfide with silver jewellery. Suicide had occurred from the inhalation of hydrogen sulfide (not ‘hydrogen cyanide’ as had been written on a warning sign) in the confined space of a vehicle cabin. Despite the absence of toxicological findings, death was attributed to hydrogen sulfide inhalation given the compelling scene findings of a strong rotten egg smell with a toxic gas registered on a detector. In addition, the history of depression, suicide note and location clearly established a suicidal motive. The presence of brick, tile and paver cleaner and lime sulfur demonstrated the origin of the hydrogen sulfide gas with its characteristic rotten egg smell. In addition there were no competing causes of death with no evidence of trauma and no underlying natural diseases which could have caused death. The green discoloration of only the brain is also strongly supportive of hydrogen sulfide toxicity.

Hydrogen sulfide is a colorless gas that has a smell of rotten eggs (noted at the above scene) or cabbage. Inhalation may be lethal with fatalities most often arising in industrial settings such as those that involve petroleum, gas or sewage processing where organic material has decomposed. It is heavier than air and so poses a particular problem in mines and sewers [1]. While carbon monoxide is the most common cause of lethal workplace gas inhalation (at 36%), hydrogen sulfide ranks second (at 7.7%) [2]. However, as with carbon monoxide, hydrogen sulfide may also be used in cases of suicide, although less commonly [3, 4].

Hydrogen sulfide suicides began in Japan where over five hundred deaths occurred in early 2008 after it was first publicized on internet sites in 2007 that detailed how to obtain the required commercial ingredients [1, 5, 6]. In the United States 2 cases in 2008 had increased to 18 by 2010 [4]. Similar trends have occurred in suicides utilizing inert gases due to dissemination of information over the internet [7].

The method of generating hydrogen sulfide involves combining readily-available sulfur-containing household chemicals/cleaners or bath salts with acid cleaners, thus leading to the name ‘detergent suicide’ [6, 8]. In the current case a cleaner containing sulfamic and hydrochloric acids was mixed with lime sulfur which contains calcium polysulfides. The toxicity of hydrogen sulfide results from its bonding with ferric ions in mitochondrial cytochrome oxidase preventing cellular metabolism, a manner of action similar to that of cyanide toxicity [1].

Rapid loss of consciousness and death can occur with exposure to high concentrations (> 500ppm), with sudden loss of consciousness from hydrogen sulfide inhalation being referred to as the ‘knockdown’ effect [6]. The toxicity is demonstrated by cases of secondary contact in individuals who have been exposed to patients/bodies who have inhaled hydrogen sulfide, causing them to complain of watering eyes, headaches and dizziness. This has been estimated to occur in 80% of bystanders or first responders. Environmental contamination may persist for 12 to 37 h [4, 6, 9]. It has been suggested that its availability and lethality make it an attractive agent for use by terrorist groups [8].

At autopsy, nonspecific pulmonary and cerebral edema are noted along with variable greenish discoloration of the skin, airways, lungs, liver, esophagus, stomach and brain [10, 11]. The cause of the green discoloration, which may be limited only to the brain (as in the current case), is unclear, but may be related to denaturation of sulfur and hemoglobin [12, 13]. Problems occur with post mortem determination of levels of hydrogen sulfide which is unstable and which is also produced in decomposing bodies. Although thiosulfate has been regarded as a preferred post mortem test to confirm hydrogen sulfide exposure, as it is more stable, levels have varied considerably as they are affected by environmental concentrations of hydrogen sulfide and the time from exposure to death [14]. For example, it has been noted that very high levels of environmental hydrogen sulfide may cause almost instantaneous death, thus precluding transit to the liver and metabolism to thiosulfate [15].

Finally, an observation of note in the current case was black tarnishing of a silver necklace and silver ring, with staining of the underlying skin. Although silver is relatively nonreactive, in the presence of hydrogen sulfide gas it will develop black tarnish due to the formation of silver sulfide (Ag_2_S) [16]. We are not aware of any other reports of skin discoloration in the forensic literature from silver jewellery exposed to hydrogen sulfide, and suggest that this may be a subtle external marker to add to other findings in cases of lethal or non-lethal hydrogen sulfide exposure. This finding may be of particular use in focussing investigations if hydrogen sulfide exposure has not been suspected.

## Data Availability

There is no original data other than the case details.
